# Right ventricle speckle tracking in bronchopulmonary dysplasia: one-year follow-up

**DOI:** 10.1186/s43044-023-00336-7

**Published:** 2023-02-09

**Authors:** Marcos Clavero-Adell, Daniel Palanca-Arias, Marta López-Ramón, Lorenzo Jimenez-Montañés, Itziar Serrano-Viñuales, Segundo Rite-Gracia, Ariadna Ayerza-Casas

**Affiliations:** 1 Pediatric Cardiology Department, Miguel Servet University Hospital, Paseo Isabel La Católica 1-3, 50009 Saragossa, Spain; 2 Dislipemias Primarias, IIS Aragón, CIBERCV, Saragossa, Spain; 3 Neonatal Care Department, Miguel Servet University Hospital, Paseo Isabel La Católica 1-3, 50009 Saragossa, Spain

**Keywords:** Right ventricle strain, Speckle tracking, Echocardiography, Bronchopulmonary dysplasia, Preterm

## Abstract

**Background:**

Bronchopulmonary dysplasia (BPD) is still a major concern in preterm infants and adequate screening methods for secondary right ventricle (RV) failure are needed. Early detection could be aided by taking measurements of RV deformation using speckle tracking echocardiography. A prospective longitudinal study was carried out over 28 months at a tertiary care pediatric cardiology center. Preterm infants < 32 weeks gestational age (GA) were eligible for the study. Enrolled patients were separated in two groups: NO-BPD or BPD. At three timepoints over the first year of life, echocardiogram measurements were performed. Right ventricle strain was studied using speckle tracking analysis and compared to conventional function parameters.

**Results:**

Fifty patients were enrolled in the study, 22 in the NO-BPD group and 28 in the BPD group. RV strain showed no statistical differences between groups. However, the BPD group showed worse RV function than the NO-BPD group, using speckle tracking analysis and other conventional parameters. During the study follow-up, an improvement trend is shown in RV strain.

**Conclusions:**

RV longitudinal strain and strain rate derived by speckle tracking is feasible in preterm infants. Although there seems to be a good correlation between RV strain and BPD severity, the results of this study were not conclusive. More studies should be carried out to investigate the optimum echocardiographic screening model of RV dysfunction in BPD patients.

**Supplementary Information:**

The online version contains supplementary material available at 10.1186/s43044-023-00336-7.

## Background

Perinatal medicine has experienced many advances over the last two decades. Despite this, bronchopulmonary dysplasia (BPD) is still one of the most common and worrying diseases in the preterm newborn [1]. BPD is defined as oxygen need for ⩾28 days from birth until 36 weeks of postmenstrual age (PMA). In 2000, BPD was categorized as none, mild, moderate or severe. None BPD (NO-BPD) was defined as < 28 days of oxygen therapy. Mild BPD (MI-BPD) included preterm newborns who received oxygen or mechanical respiratory support for > 28 days but were self-ventilating by 36 weeks PMA. Moderate BPD (MO-BPD) is referred to the requirement of supplemental oxygen, but < 30% fraction of inspired oxygen concentration, at 36 weeks PMA. Finally, severe BPD (SE-BPD) included those patients who required > 30% oxygen or positive pressure at 36 weeks PMA [2, 3]. More recent investigations have tried to establish a more accurate definition of BPD to predict childhood outcomes in modern-day very preterm infants [4].

BPD indicates the presence of perturbations on the lung alveolar septation with varying degrees of lung fibrosis and inflammation. BPD is usually associated with abnormalities in lung microvascular development, which may contribute to the modified alveolar septation. Furthermore, lung capillary and arterioles are usually affected in BPD patients, with thicker-walled pulmonary arteries. In view of this, it is possible to consider that BPD patients not only suffer from alterations in lung structure and function but also may have increased vascular resistance and pulmonary arterial pressures [5]. Therefore, echocardiogram screening methods strategies for pulmonary hypertension (PH) and right ventricle (RV) dysfunction are indicated during early childhood [6, 7].

RV myocardial deformation during the cardiac cycle can be studied through strain analysis. 2D speckle tracking (STE) is a recent application software for strain study. STE imaging provides frame-by-frame tracking of ultrasound markers, is angle independent, is less sensitive to signal noise, and is not influenced by breathing movement or by the interaction with the adjacent myocardium. This technique has enabled earlier detection of subtle RV dysfunction [8]. The aim of this study was to assess the applicability/ feasibility of using this analysis method in a cohort of preterm patients followed up longitudinally over the first year of life and to determine its clinical value as a screening tool in patients with BPD.

## Methods

### Study design and patients

This prospective longitudinal study was carried out over 28 months (from April 2019 to July 2021) at a tertiary care pediatric cardiology referral center. All preterm infants (PTI) < 32 weeks who were born before July 2020 were eligible for the study. Twenty-eight days after birth, all enrolled patients were included in one of two groups: no bronchopulmonary dysplasia (NO-BPD) or bronchopulmonary dysplasia (BPD). They were screened during routine follow-up at a tertiary care pediatric referral center, which routinely includes several cardiac evaluations. Follow up data was collected at three timepoints: at 36 PMA or discharge (T1), between 5 and 9 months of life (T2), and between 11 and 16 months of life (T3) [9]. When echocardiography studies were performed, patients were at basal conditions, they had no disturbances which could interfere with the results (e.g., sepsis, patent ductus arteriosus or respiratory infections). Anthropometric data (weight, height), medical history/background and echocardiographic measurements were documented. Patients affected from congenital heart disease, major genetic disorders or cardiomyopathy were excluded.

### Conventional echocardiography

Echocardiographic examinations were performed using a Siemens Acuson SC2000 ultrasound system (Siemens Healthcare, Erlangen, Germany) equipped with an 8 MHz sector transducer. Two pediatric cardiologists received the same training to perform all the echocardiographic examinations, following current guidelines [10]. Image acquisition procedures were harmonized before the study started. Optimal frame rate (60 to 92 frames/sec) was used to optimize myocardial deformation analysis. ECG-guided, we systematically recorded three cardiac-cycle loops in the following views: apical classic view, apical focused on right ventricular (RV)-free wall and shot-axis view focused on RV outflow tract (RVOT) and pulmonary trunk. We also used pulse-wave Doppler, tissue Doppler and M-mode to analyze cardiac function and heart flows. The following conventional RV function variables were measured: Right atrium area indexed by body surface (RA index), tricuspid annular plane systolic excursion (TAPSE), Doppler tissue imaging tricuspid S wave velocity (SW), RV fractional shortening (RV-FS), pulmonary artery acceleration time (PAAT), right ventricular ejection time (RVET), tricuspid regurgitation pressure gradient (TRPG) and right atrium area (RA) [11]. Systolic pulmonary pressure was estimated by TRPG.

### Speckle tracking (STE) analysis

**Fig. 1:**
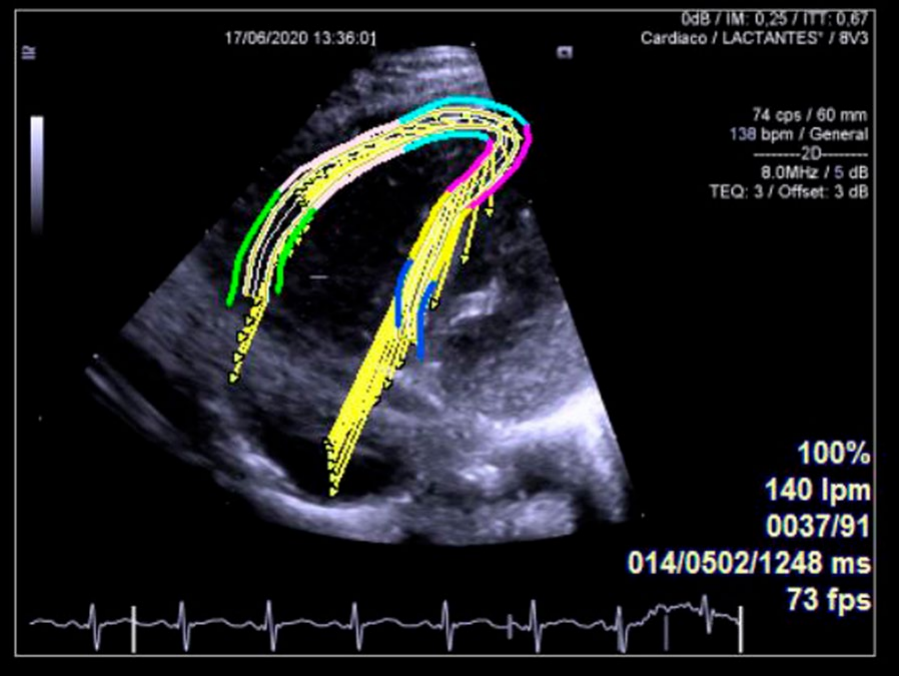
Right ventricle-focused four chamber view. Strain analysis of right ventricle with speckle tracking software. The endocardial and epicardial borders were manually delineated to perform a semiautomatic trace of the myocardial movement. The magnitude and direction of each vector reflect the corresponding myocardial movement towards the reference point

Both sonographers performed offline strain analysis on apical loops (classic and RV-focused) using the software Velocity Vector Imaging (VVI) 3.0 (Siemens). A second analysis was performed on a sample of 10 randomly selected subjects to assess intra and interobserver reproducibility with no access to the results of the first analysis. The investigators manually traced the endocardium in end-diastole. The software detected the movement of the entire myocardial wall (from the endocardium to the epicardium) and therefore defined the areas of interest, for which the quality was considered acceptable or not (Fig. 1). VVI software automatically splits the ventricular wall into 6 segments. For measuring LV strain, the whole six segments were used. For measuring RV strain, only the three RV free wall segments were used (basal, mid and apical). In poorly detected segments, the sonographer readjusted the endocardial contour until better detection was obtained. Whenever that was not possible two rejection methods were defined: For LV, the segments in question were excluded from the analysis and the rest were used (maximum of two segments); for RV, STE data were excluded. We measured the following global STE variables: global systolic LV longitudinal strain (GLS-LV) and strain rate (GLSR-LV), global systolic RV longitudinal strain (GLS-RV) and strain rate (GLSR-RV). Strain data were shown in absolute values to avoid confusion.

### Formal aspects

The study was conducted in compliance with the Good Clinical Practices protocol and Declaration of Helsinki principles. Approval was granted by Clinical Research Ethics Committee of Aragón (CEICA). Informed consent was obtained from all parents or legal guardians.

### Statistical analysis

SPSS software version 25.0 (SPSS Inc., Chicago, IL, USA) was used for performing statistical analysis. Any difference was considered statistically significant in all tests when the *P* value was less than 0.05. To determine normality, the distribution of the longitudinal strain and the longitudinal strain rate of both ventricles in all subjects was assessed using the Kolmogorov–Smirnov's and Shapiro–Wilk's test. Summary results of the main measures are shown for each group. Continuous variables were described by the mean and standard deviation (SD), or by median and interquartile range [Q1–Q3] if the variable did not meet the assumptions for data normality. Categorical variables were expressed by absolute frequency as well as percentage. The association between parametric variables was measured using Pearson correlation and for non-normal variables, the spearman correlation coefficient instead. For assessed normal samples, mean differences were tested by t-Student test. In other cases, U-Mann–Whitney test was performed. Pearson's Chi-square test was used for comparisons between categorical variables. The relationship with time was determined by the Friedman test. The reproducibility of GLS measurements was assessed using intra and interobserver reliability using 10 blinded images randomly sampled from the study. Intraobserver reliability was good, with an intraclass correlation coefficient (ICC) above 0.77. Interobserver reliability was relatively good, with an ICC above 0.69.

## Results

Fifty patients were enrolled in the study, 22 in the NO-BPD group and 28 in the BPD group (16 mild, 8 moderate, 4 severe). Characteristics are revealed in Table 1. We found some demographic differences such as birth weight and weeks of pregnancy at birth between the two groups. As expected, BPD group required more ventilatory assistance during intensive care unit (ICU) admission. As personal background, they also suffered sepsis (vertical and nosocomial) and significant arteriosus ductus more frequently, receiving proper treatment according to guidelines. This last issue was tested not to be related to some of the ultrasound measurements made at T1, T2, T3 (Additional file 1). A larger number of patients in the BPD group suffered bronchiolitis during the first year of life. It was also observed that in the BPD group, 6 patients suffered more than one acute respiratory failure around this period. No patient required home ventilation therapy.

**Table 1:** Sample characteristics for both groups

	NO-BPD (*n* = 22)	BPD (*n* = 28)	*p* value
Birth weight (mea*n*; g)	1412	840	0.000
Small for gestational age (*n*)	1 (4.5%)	6 (21.4%)	0.088
Weeks of pregnancy at birth (median; weeks + days)	31 + 4	26 + 3	0.000
Multiple gestation (*n*)	10 (45.5%)	13 (46.4%)	0.945
Complete fetal lung maturation (*n*)	3 (13.6%)	4 (14.3%)	0.526
Chorioamnionitis (*n*)	1 (4.5%)	8 (28.6%)	0.028
Advanced neonatal resuscitation (n)^a^	0	3 (10.7%)	0.128
Apgar 1 (median)	9	6	0.000
Apgar 5 (median)	10	8	0.000
Surfactant (*n*)	2 (9.1%)	25 (83.9%)	0.000
More than one surfactant dose (*n*)	2 (9.1%)	14 (58%)	0.000
Significant patent ductus arteriosus which needed treatment (*n*)	3 (13.6%)	13 (46.4%)	0.014
Invasive mechanical ventilation (IMV) (*n*)	3 (13.6%)	18 (64.3%)	0.000
Days of IMV (median)	0	1	0.000
Nitric-oxide after birth (n)	0	3 (10.7%)	0.113
Days of noninvasive mechanical ventilation (median)	5	35	0.000
Days of low-flow oxygen therapy before discharge (median)	0	14	0.000
Necrotizing enterocolitis (*n*)	2 (9.1%)	8 (28.6%)	0.087
Vertical sepsis (*n*)	0	6 (21.4%)	0.021
Nosocomial sepsis (*n*)	2 (9.1%)	18 (64.3%)	0.000
Days of diuretic treatment (median)	0	27	0.000
Days of parenteral nutrition (median)	4	14	0.000
Length of admission (mean)	43	86	0.000
Home oxygen therapy (*n*)	0	5 (17.9%)	0.037
Home ventilation therapy (*n*)	0	0	
Bronchiolitis during first year of life (*n*)	2 (9%)	11 (39.2%)	0.016
More than one acute respiratory failure (*n*)	1 (4.5%)	6 (21.4%)	0.021

Scalar variables are shown as mean/median. Nominal variables are shown as the number of patients (*n*) and percentage. T-Student test was calculated for parametric variables. Kruskal–Wallis test for nonparametric variables. Pearson's Chi-square test for categorical variables

^a^More than noninvasive mechanical ventilation

Ultrasound parameters of cardiac function were measured. The results are expressed as mean and standard deviation, or median and interquartile range (Table 2). At T1, fifty measures of each variable were achieved. At T2, 4 patients did not attend to the medical appointment (2 moved to a different city and 2 refused because of the COVID pandemic). At T3, it was not possible to perform good quality images in 11 patients. T-Student or U-Mann–Whitney test were performed to analyze differences between groups. No statistical differences were found. However, at the three timepoints, the data from the BPD showed worse RV function, with lower TAPSE, SW, RV-FS and RV strain. BPD group also showed lower LV strain values at T1 than NO-BPD group (no statistical significance). This finding was not associated with the presence of significant patent ductus arteriosus. These differences disappeared at T2 and T3. None of the patients suffered from pulmonary hypertension, defined by an increased estimated systolic PAP more than 40% of systemic arterial pressure. However, at T1 and T3, there were differences between groups in TRPG.

**Table 2:** Echocardiographic measurements at the three timepoints

	Mean (SD) or Median [Q1; Q3]		*p* value
	NO-BPD (*n* = 22)	BPD (*n* = 28)	
T1			
HR (bpm)	164.5 (13.6)	164.8 (12.5)	0.942
RA index (cm^2^/m^2^)	9.1 (1.6)	9.4 (2.1)	0.493
TRPG (mmHg)	14 (8)	20 (7)	0.006
TAPSE (mm)	10.1 (1.7)	9.7 (1.7)	0.378
SW (cm/s)	9.8 [9; 11.7]	9.9 [9; 11.7]	0.537
PAAT / RVET ratio	0.34 (0.09)	0.33 (0.08)	0.714
GLS-RV (%)	23.9 (4.6)	22 (6.3)	0.215
GLSR-RV (%)	2.62 [2.26; 3.16]	2.33 [1.92; 2.67]	0.062
RV-FS (%)	47.3 [39.4; 51.5]	46.3 [34; 49.8]	0.226
GLS-LV (%)	22 (3.2)	20.4 (3.3)	0.094
GLSR-LV (%)	2.23 (0.51)	2.07 (0.41)	0.224

	NO-BPD (*n *= 21)	BPD (*n* = 25)	
T2			
HR (bpm)	138.9 (18.1)	145.7 (14.2)	0.176
RA index (cm^2^ / m^2^)	8.8 [7.6; 9.5]	8.8 [7.5; 9.5]	0.384
TRPG (mmHg)	13 (7)	17 (10)	0.180
TAPSE (mm)	14.8 (2.1)	13.4 (2.4)	0.039
SW (cm/s)	12.2 (1.7)	11.2 (1.6)	0.064
PAAT / RVET ratio	0.38 (0.07)	0.39 (0.08)	0.401
GLS-RV (%)	26 [21.2; 28.2]	24.1 [18.9; 27.9]	0.349
GLSR-RV (%)	2.57 (0.88)	2.43 (0.82)	0.594
RV-FS (%)	39.3 (13.7)	42.1 (13.5)	0.500
GLS-LV (%)	22.8 (3.5)	22.8 (3.5)	0.995
GLSR-LV (%)	2.3 (0.49)	2.07 (0.55)	0.157

	NO-BPD (*n* = 15)	BPD (*n *= 16)	
T3			
HR (bpm)	131 (16.6)	128.7 (17.6)	0.699
RA index (cm^2^/m^2^)	8.3 (1.6)	9.9 (1.7)	0.007
TRPG (mmHg)	9 (16)	15 (6)	0.011
TAPSE (mm)	15.7 [13.8; 16.7]	14.7 [14.5; 15.9]	0.717
SW (cm/s)	12.3 (2.1)	12.1 (1.6)	0.806
PAAT / RVET ratio	0.37 (0.06)	0.41 (0.07)	0.05
GLS-RV (%)	28 (6.1)	26 (4.1)	0.315
GLSR-RV (%)	2.88 (0.85)	2.60 (0.75)	0.335
RV-FS (%)	43.6 [35.6; 56.9]	41.7 [32.5; 46.4]	0.166
GLS-LV (%)	23.8 (4.5)	24.4 (3.1)	0.671
GLSR-LV (%)	2.28 [1.96; 2.57]	2.07 [1.76; 2.38]	0.349

Classification in NO-BPD and BPD. Mean (Standard Deviation) for parametric variables and T-Student test for comparison between groups (*p* value). Median [interquartile range Q1; Q3] for nonparametric variables and U-Mann–Whitney test for comparison between groups (*p* value).

HR: heart rate. BPM: beats per minute. T1: at 36 PMA or discharge. T2: between 5 and 9 months of life. T3: between 11 and 16 months of life.

**Fig. 2:**
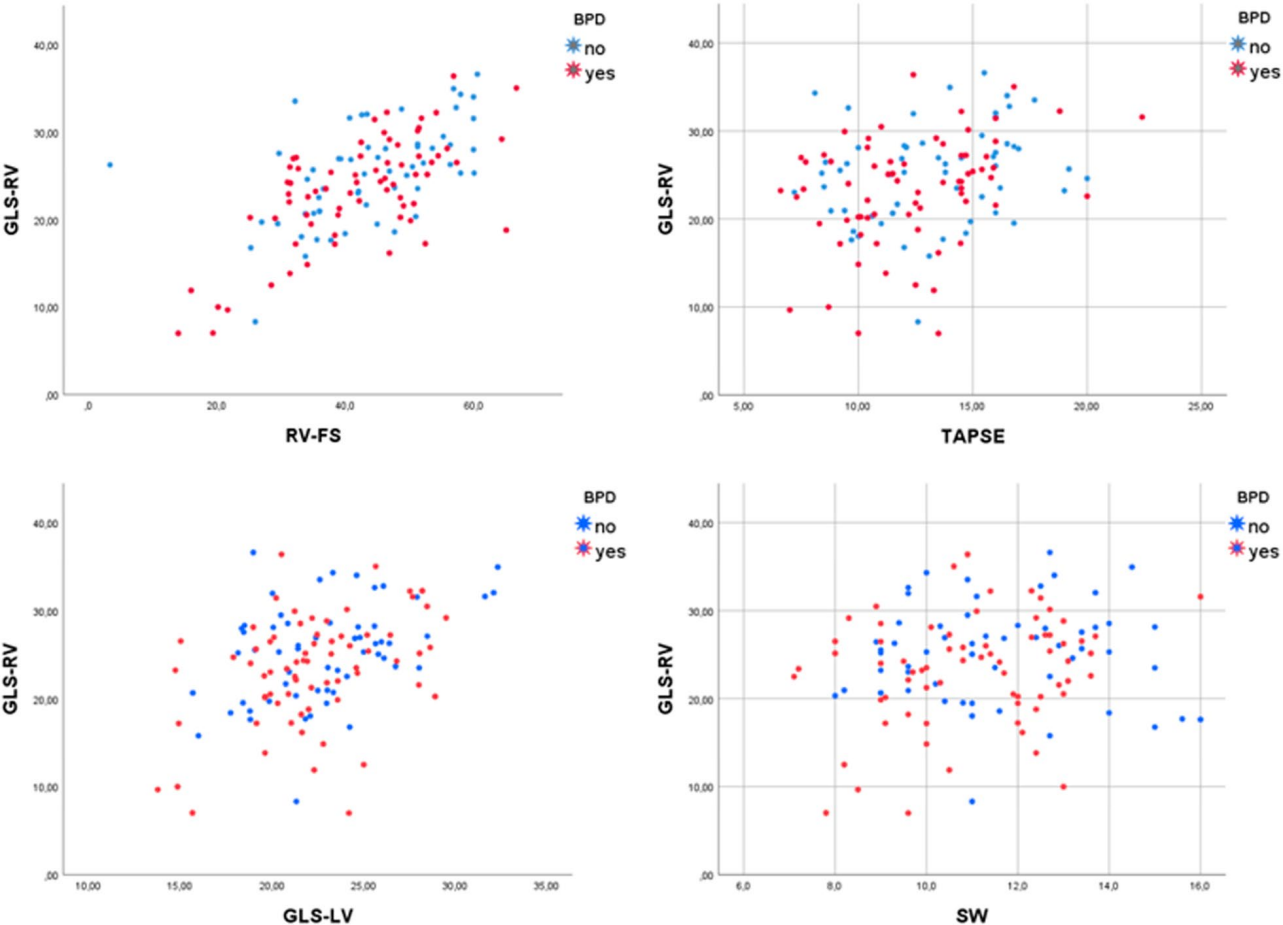
Scatter plot relating GLS-RV with RV-FS, TAPSE, GLS-LV and SW. It shows clear positive correlations between GLS-RV and RV-FS or GLS-LV. There is a possible positive trend between GLS-RV and TAPSE. No correlation is observed between GLS-RV and SW

Pearson test was used to study association between variables at the three timepoints. It showed significant good positive correlation between GLS-RV vs. RV-FS (*R* = 0.617) and between GLS-RV vs. GLS-LV (*R* = 0.403). A small positive correlation was found between GLS-RV vs. TAPSE (*R* =  + 0.303). No correlation was found between GLS-RV and SW (Fig. 2).

**Fig. 3:**
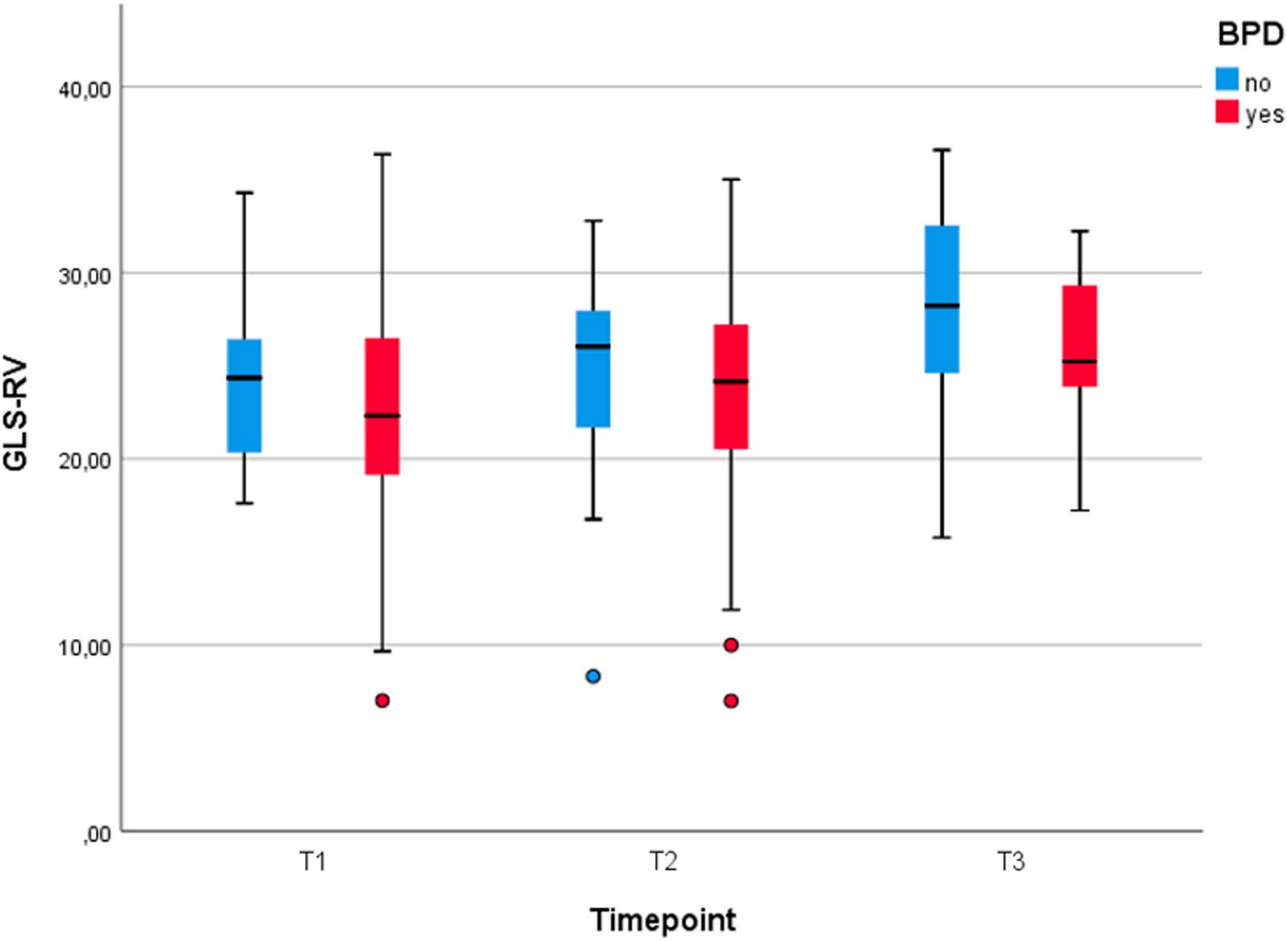
GLS-RV (mean and standard deviation) at different timepoints. Although it is no significant, we can appreciate that GLS-RV tends to improve along time, which is more evident in BPD patients

**Fig. 4:**
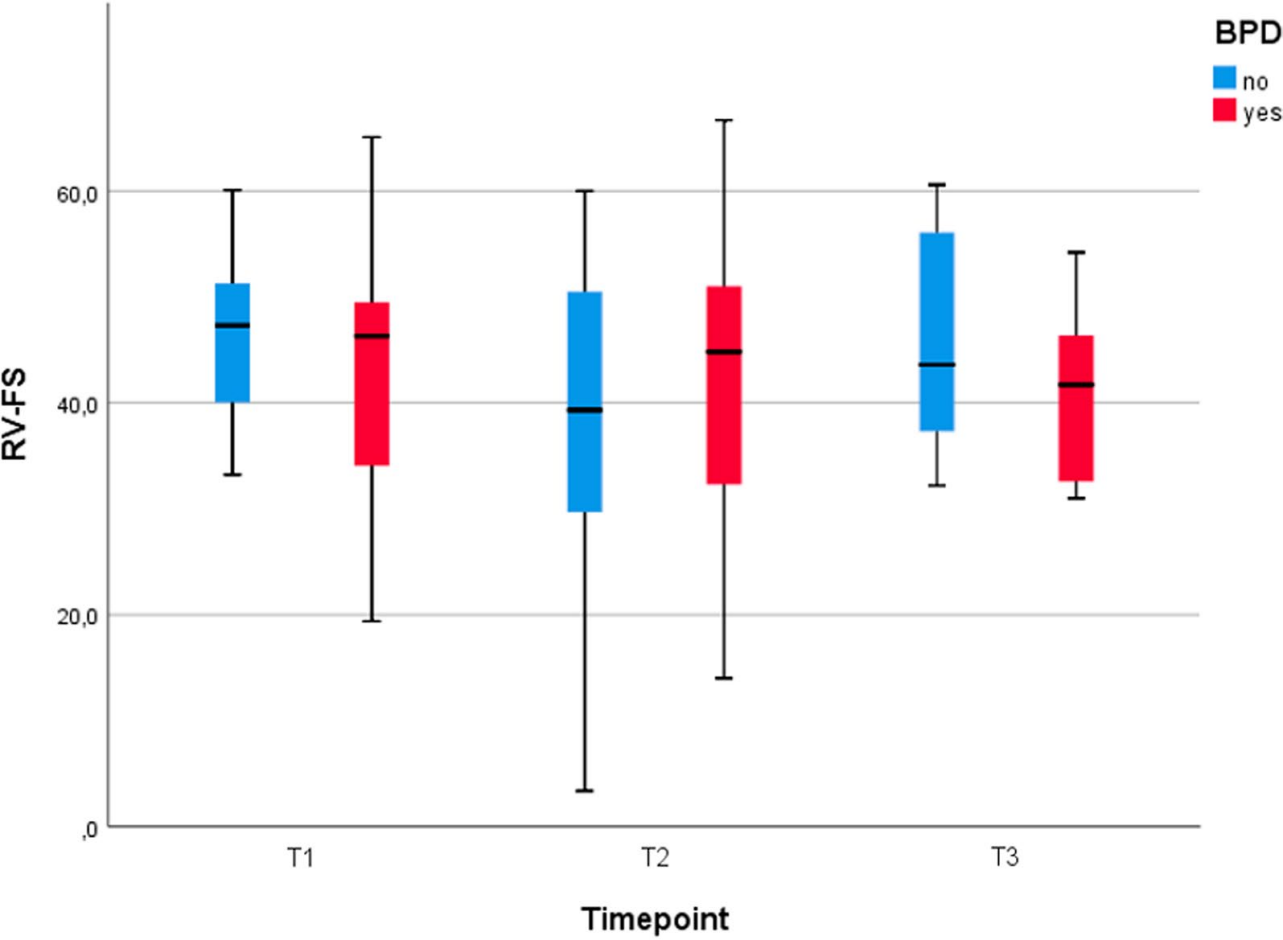
RV-FS (mean and standard deviation) at different timepoints. No clear changes in RV-FS can be seen with time in either group (BPD or NO-BPD patients)

Friedman test was used to study cardiac function variables along time (T1, T2, T3). No differences were found in strain variables of both ventricles (GLS, GLSR), RV-FS, RA index and PAAT / RVET ratio. However, an improvement trend is shown in GLS-RV (Fig. 3) and GLSR-RV. Other variables of RV function, like RV-FS, did not show it (Fig. 4). RV strain measurements did not show a relationship with the duration of the respiratory support or the incidence of bronchiolitis over the first year of life either.

## Discussion

BPD is a chronic lung disease that, with the influence of multiple factors, generates lung alterations not only in the airway but also in the pulmonary vessels, leading to a variable worsening of cardiopulmonary function. Recent advances in perinatal medicine have decreased its incidence, but BPD is still responsible for significant morbidity of preterm infants [3]. PH is a rare complication of BPD, but it is known that the appearance of PH worsens the clinical course, morbidity and mortality associated with BPD [12]. The gold standard technique for the diagnosis of PH is by cardiac catheterization, an invasive procedure requiring general anesthesia in young children. It allows a direct measurement of the pulmonary arterial pressure (PAP). However, transthoracic echocardiography is more commonly used in children for its ability to estimate PAP and its consequences on RV [12]. Mourani et al. (2008) did a retrospective review of data from 25 infants who underwent echocardiography and subsequent cardiac catheterization for the evaluation of pulmonary hypertension. Compared with cardiac catheterization, echocardiography had 79% sensitivity of detecting the presence of pulmonary hypertension [14].

Several traditional echocardiographic measurements are usually used for screening PH. Tricuspid regurgitation pressure gradient (TRPG) represents the most common and reliable method to evaluate the presence and severity of PH [6]. In our study, TRPG was used to assess the presence of PH. Other echocardiographic methods have been studied to analyze RV function in preterm infants. Sehgal et al. assessed RV function using tissue Doppler imaging (TDI), 2D RV-FS, TAPSE, and myocardial performance index (MPI) using echocardiography. They found that higher E/e´ and lower RV-FS showed strong correlations with the subsequent duration of respiratory support during hospitalization, although the data was within normal values. The rest of parameters had no relevance [15]. We did not find correlation between the different echocardiographic methods and the duration of ventilatory support. However, there is lack of evidence in the literature regarding the echocardiographic management of these patients.

Ventricular function derivates from the contraction and relaxation of a complex architecture of myocardial fibers. As a result, the assessment of myocardial deformation by two-dimensional speckle tracking echocardiography gained greater acceptance. It has been proved that strain measurements can be used as an early marker of cardiac dysfunction. It is also known that RV strain is a feasible technique [8, 16]. RV strain predicts mortality in a population of stable patients with chronic heart failure with reduced LV ejection fraction [17], predicts the prognosis after acute myocardial infarction in adults [18] or mortality in patients with COVID-19 [19]. RV strain has also been a valuable tool for the evaluation of RV in PH patients with several etiologies [20, 21]. Therefore, in this research the authors decided to assess the usefulness of GLS-RV in patients who suffered from lung disease but without pulmonary hypertension. We hypothesized that BPD could modify the lung architecture, increasing the stiffness of pulmonary arterioles and causing pressure overload (without PH). Strain measurements could help us to discover a “hidden” RV dysfunction.

Xie et al. evaluated strain in children between 3 and 5 years old. They found some differences in RV strain between preterm BPD patients and term infants, and that duration of invasive ventilation was independently linked with GLS-RV [22]. In our study, we compared the BPD with NO-BPD patients, but all of them were PTI. Perhaps, GLS impairment is associated not only with BPD, but also with prematurity.

Haque et al. also studied RV function in BPD patients. They did not find differences in traditional echocardiographic parameters, but using speckle tracking they discovered that infants with severe BPD had lower peak global systolic strain than infants with moderate BPD or mild/none BPD [23]. However, other authors did not find differences between the BPD and NO-BPD groups using either traditional echocardiographic parameters or through myocardial deformation analysis [24].

Blanca et al. designed a similar study to ours [25]. They included BPD patients. Their sample involved patients with and without PH. At 6 months PMA, they found differences in RV fractional shortening and GLS-RV between non-PH and PH patients (all of them had BPD). In our investigation, we did not find PH, hoping that GLS-RV would contribute to detect subclinical changes in RV myocardial damage. However, it is likely that larger variations in clinical presentation are needed to find significant differences in GLS. Our cohort also shows an improvement with time, which supports the theory that patients with non-severe clinical conditions (non-PH patients) demonstrate a total recovery of myocardial alterations.

As for the GLS-LV data in the BPD group at T1, we propose a correlation with RV function. When echocardiographic measurements were taken, the septum took part in LV strain so RV movement may be related to these findings. Czernik et al. (2014) studied LV strain over the first month of life in BPD patients. They found higher values of LV strain in the BPD group during the first two weeks of life, which disappeared at one month of life. They suggest their findings reflect the hemodynamic changes that appear within the first days of life and the volume overload that generates a patent ductus permeable [26]. In our cohort, the first analysis was done at 36 weeks PMA and no relationship was found with the presence of patent ductus arteriosus (PDA). However, at this timepoint, PDA-associated problems are usually resolved.

This study has several strengths and limitations. The main strengths include the longitudinal follow-up over the first year of life and the standardized collection of data. We used the same equipment and protocol for all patients and echocardiographic images and measurements were made by the same investigators. The study was limited by offline speckle tracking analysis. The dependence of 2D-STE imaging on the frame-by-frame tracking of the myocardial pattern means it is influenced by image factors, including reverberation artefacts and attenuation. Thus, we lost too many cases at T3, due to the requirement of a very good loop imagen to complete a reliable strain analysis. Our study was also limited by the small sample size which makes difficult to find significant differences and generates large variances. It is possible that incorporating patients with PH in this study may have given further insight into the clinical presentation of patients with BPD.

## Conclusions

Our study demonstrates that, even though it is challenging, measuring RV longitudinal strain and strain rate derived by speckle tracking is feasible in preterm infants. Although the authors of this study did not find any statistical differences between the groups, good correlation between RV strain and BPD severity was observed. More studies should be carried out to investigate the optimum echocardiographic screening model of RV dysfunction in BPD patients (including strain measurements or not) and to confirm that non-PH patients who suffered from BPD maintain normal RV function with age.

## Supplementary Information

**Additional file 1.** Echocardiographic measurements at the three timepoints. Classification according to the presence of a significant patent ductus arteriosus (PDA) which needed treatment (drugs or surgery). Mean (Standard Deviation) for parametric variables and T-Student test for comparison between groups (p value). Median [interquartile range Q1; Q3] for non-parametric variables and U-Mann-Whitney test for comparison between groups (p value).

## Data Availability

The datasets used and/or analyzed during the current study are available from the corresponding author on reasonable request.

## Abbreviations

BPD: Bronchopulmonary dysplasia
ECG: Electrocardiography
GLS: Global systolic longitudinal strain
GLSR: Global systolic longitudinal strain rate
ICC: Intraclass correlation coefficient
ICU: Intensive care unit
LV: Left ventricle
MPI: Myocardial performance index
PAAT: Pulmonary artery acceleration time
PAP: Pulmonary arterial pressure
PDA: Patent ductus arteriosus
PH: Pulmonary hypertension
PMA: Postmenstrual age
PTI: Preterm infants
RA: Right atrium
RV: Right ventricle
RVET: Right ventricular ejection time
RV-FS: Right ventricle fractional shortening
RVOT: Right ventricle outflow tract
SD: Standard deviation
STE: Speckle tracking
SW: Doppler tissue imaging tricuspid S wave velocity
TAPSE: Tricuspid annular plane systolic excursion
TRPG: Tricuspid regurgitation pressure gradient
VVI: Velocity vector imaging
