# Supplementary material for: Right ventricle speckle tracking in bronchopulmonary dysplasia: one-year follow-up
Source: Egypt Heart J. 2023 Feb 9;75:10. doi: 10.1186/s43044-023-00336-7 (PMC9909144; doi:10.1186/s43044-023-00336-7)
Supplement: Supplementary file 1 — Additional file 1. Echocardiographic measurements at the three timepoints. Classification according to the presence of a significant patent ductus arteriosus (PDA) which needed treatment (drugs or surgery). Mean (Standard Deviation) for parametric variables and T-Student test for comparison between groups (p value). Median [interquartile range Q1; Q3] for non-parametric variables and U-Mann-Whitney test for comparison between groups (p value). [file 43044_2023_336_MOESM1_ESM.docx]

Supplementary material.

Echocardiographic measurements at the three timepoints. Classification according to the presence of a significant patent ductus arteriosus (PDA) which needed treatment (drugs or surgery). Mean (Standard Deviation) for parametric variables and T-Student test for comparison between groups (p value). Median [interquartile range Q1; Q3] for non-parametric variables and U-Mann-Whitney test for comparison between groups (p value).

|  | | Mean (SD) or Median [Q1; Q3] | | p value |
| --- | --- | --- | --- | --- |
|  | | NO-PDA (n = 34) | PDA (n = 16) |  |
|  | RA index (cm2/m2) | 9.4 (1.8) | 9.1 (2.2) | 0.641 |
|  | TRPG (mmHg) | 16 (8) | 19 (7) | 0.108 |
|  | TAPSE (mm) | 9.9 (1.8) | 9.8 (1.5) | 0.916 |
|  | SW (cm/s) | 10 [9.2; 12] | 9 [8.2; 11.1] | 0.080 |
|  | PAAT / RVET ratio | 0.33 (0.09) | 0.33 (0.09) | 0.252 |
|  | GLS-RV (%) | 22.6 (6.2) | 23.5 (4.5) | 0.608 |
|  | GLSR-RV (%) | 2.38 [1.95; 2.9] | 2.38 [1.99; 3.1] | 0.950 |
|  | RV-FS (%) | 46.6 [34,5; 52.5] | 46.5 [42.4; 49.8] | 0.925 |
|  | GLS-LV (%) | 21.1 (3.8) | 21 (2.3) | 0.935 |
|  | GLSR-LV (%) | 2.17 (0.50) | 2.10 (0.35) | 0.613 |
|  | | NO-PDA (n = 32) | PDA (n = 14) |  |
|  | RA index (cm2 / m2) | 8.9 [7.5; 10.2] | 8.7 [7.5; 10] | 0.962 |
|  | TRPG (mmHg) | 15 (9) | 16 (10) | 0.547 |
|  | TAPSE (mm) | 14.3 (2.6) | 13.3 (1.6) | 0.181 |
|  | SW (cm/s) | 11.7 (1.8) | 11.4 (1.6) | 0.643 |
|  | PAAT / RVET ratio | 0.39 (0.07) | 0.37 (0.07) | 0.446 |
|  | GLS-RV (%) | 24.5 [20.8; 28.4] | 25.2 [19.1; 27.5] | 0.738 |
|  | GLSR-RV (%) | 2.51 (0.84) | 2.45 (0.88) | 0.820 |
|  | RV-FS (%) | 40.5 (13.7) | 41.6 (13.6) | 0.800 |
|  | GLS-LV (%) | 22.9 (3.5) | 22.6 (3.5) | 0.766 |
|  | GLSR-LV (%) | 2.23 (0.55) | 2.06 (0.59) | 0.318 |
|  | | NO-PDA (n = 23) | BPD (n = 10) |  |
|  | RA index (cm2/m2) | 8.8 (1.8) | 9.9 (1.6) | 0.097 |
|  | TRPG (mmHg) | 11 (8) | 16 (3) | 0.113 |
|  | TAPSE (mm) | 15.6 [14; 16.8] | 14.7 [14.5; 15.9] | 0.726 |
|  | SW (cm/s) | 12.1 (1.6) | 12.3 (2.3) | 0.782 |
|  | PAAT / RVET ratio | 0.38 (0.07) | 0.42 (0.06) | 0.129 |
|  | GLS-RV (%) | 26.7 (5.7) | 27.6 (3.7) | 0.661 |
|  | GLSR-RV (%) | 2.84 (0.85) | 2.48 (0.63) | 0.263 |
|  | RV-FS (%) | 42.8 [34.7; 51.6] | 40.7 [34.9; 53] | 0.761 |
|  | GLS-LV (%) | 23.3 (3.9) | 25.9 (3.1) | 0.078 |
|  | GLSR-LV (%) | 2.15 [1.72; 2.5] | 2.26 [1.98; 2.68] | 0.531 |

T1: at 36 PMA or discharge. T2: between 5 and 9 months of life. T3: between 11 and 16 months of life.
